# RPFdb v2.0: an updated database for genome-wide information of translated mRNA generated from ribosome profiling

**DOI:** 10.1093/nar/gky978

**Published:** 2018-10-18

**Authors:** Hongwei Wang, Ludong Yang, Yan Wang, Leshi Chen, Huihui Li, Zhi Xie

**Affiliations:** 1 State Key Laboratory of Ophthalmology, Zhongshan Ophthalmic Center, Sun Yat-sen University, Guangzhou 510060, China; 2 Centre for Advanced Computational Solutions (C-fACS), Lincoln University, New Zealand

## Abstract

RPFdb (http://www.rpfdb.org or http://sysbio.sysu.edu.cn/rpfdb) is a public database for hosting, analyzing and visualizing ribosome profiling (ribo-seq) data. Since its initial release in 2015, the amount of new ribo-seq data has been considerably enlarged with the increasing popularity of ribo-seq technique. Here, we describe an updated version, RPFdb v2.0, which brings significant data expansion, feature improvements, and functionality optimization: (i) RPFdb v2.0 currently hosts 2884 ribo-seq datasets from 293 studies, covering 29 different species, in comparison with 777 datasets from 82 studies and 8 species in the previous version; (ii) A refined analysis pipeline with multi-step quality controls has been applied to improve the pre-processing and alignment of ribo-seq data; (iii) New functional modules have been added to provide actively translated open reading frames (ORFs) information for each ribo-seq data; (iv) More features have been made available to increase database usability. With these additions and enhancements, RPFdb v2.0 will represent a more valuable and comprehensive database for the gene regulation community.

## INTRODUCTION

Ribosome profiling is emerging as a powerful technique that enables genome-wide investigation of *in vivo* translation at sub-codon resolution (1,2). By precisely pinpointing ribosomes during translation, this technique can provide deeper insights into the composition, regulation and mechanism of translation (3–6). The discriminatory power of ribo-seq technique leads to its widespread applications in various organisms that are enabling data generation at an unprecedented scale. However, vast amounts of ribo-seq data are mostly stored in raw data format so that they cannot be easily inspected and analyzed. Furthermore, comparisons across different datasets cannot be reliably made without unified data processing and analytics. As a result, efficient storage, retrieval and management of these large amounts of publicly available ribo-seq data are urgently needed to the research community. To this end, several dedicated database of ribo-seq data have been built, including GWIPS-viz (7), a visualization tool for ribo-seq data; RiboSeqDB (8), a repository of selected human, mouse and rat ribo-seq data; TranslatomeDB (9), a collection of ribo-seq, RNC-seq and mRNA-seq data with emphasis on differential gene expression analysis, and our RPFdb (10). In addition, several databases are designed specifically for storing genomic elements detected from ribo-seq, such as sORFs.org (11) and uORFdb (12), hosting small ORFs (sORFs) and upstream ORFs respectively.

RPFdb is dedicated to host, analyze and visualize ribo-seq data, processed by a unified pipeline (10). Since the initial release of RPFdb in 2015, the volume of raw ribo-seq data in repositories such as the Sequence Read Archive (SRA) (13) has grown rapidly because of the increasing popularity of ribo-seq technique and reduction of sequencing costs. At the same time, substantial progress has been made in our understanding the what, when, where and how of protein synthesis (5,14). Moreover, a growing number of studies on the coding potential of genomes have showed that a diverse set of non-canonical translation products such as sORF-encoded micro-peptides do exist (15–18). Some of micro-peptides are even known to have important physiological functions (19–23). However, despite receiving more attentions, the full repertoire of non-canonical translation products is still unknown and waits for further exploration. The rapidly accumulating ribo-seq data provide a basis for discovering the ‘dark matter’ in the genome and understanding the components of the translation apparatus. Therefore, all these premises lead us to make timely update on RPFdb.

Herein, we present an updated version of PRFdb (v2.0) that currently hosts 2884 ribo-seq datasets from 293 studies, covering 29 different species. In line with the significant expansion of ribo-seq data available in the database, we also have made several major improvements in this release, including a refined analysis pipeline with multi-step quality control applied for improving the pre-processing and alignment of ribo-seq data, new functional modules providing actively translated ORFs information, and more web features for better database usability. Further details on these additions and enhancements made to PRFdb are described below.

## SUMMARY OF FEATURES IN THE INITIAL RPFdb

The initial release of RPFdb (v1.0) provided ribo-seq data for 777 samples from 82 studies covering 8 species, including *Arabidopsis, Caenorhabditis elegans, Drosophila, Escherichia coli, Saccharomyces cerevisiae*, zebrafish, mouse and human (Table 1). The web interface for RPFdb was constructed with three main functional modules: ‘Browse’, ‘Search’, and ‘Download’. The ‘Browse’ module provides a brief description for each ribo-seq dataset such as accession identifier, source name, reference genome and the associated paper; a graphical overview of mapping statistics, distribution of reads mapped to genomic regions and RPKM (Reads Per Kilobase per Million) values of each genomic region; and a tabular display for translation levels of the top-ranked genes. The ‘Search’ module provides two types of queries: ‘Gene query’ by HGNC symbol or Ensembl gene ID and ‘Study query’ by keywords. In response to the ‘Gene query’, the webpage returns mRNA translation represented by normalized RPKM values from different samples of different studies and an interactive JBrowse genome browser for visualizing read-alignment data stored in bam files. In response to the ‘Study query’, the webpage returns a meta-information on the resulting study and an extended statistical summary of the study. The ‘Download’ module provides tabulated summary table of gene translation in different genomic regions for each study.

**Table 1.** Comparison between RPFdb v2.0 and v1.0

	RPFdb v1.0	RPFdb v2.0
***Summary of data***		
Data source	SRA	SRA, ENA, DDBJ
No. of datasets	777	2884
No. of studies	82	293
No. of species	8	29
***Data processing***		
*Pre-processing steps*	• Quality control	• Quality control
	• Keep the first 26 nucleotides of each sequencing read	• Adapter removal
		• Low-quality sequence trimming
		• rRNA and tRNA filtration
		• Read-length selection (25–34 nt)
***Functionalities***		
Browse	• Study browser(overview of dataset-meta description and summary statistics)	• Study browser (overview of dataset-meta description, summary statistics, and quality control assessment)
		• ORF browser (overview and detailed annotation information on actively translated ORFs)
Search and visualization	• RPKM values	• RPKM values
	• Footprint coverage at different genomic regions	• ORF entry
		• Footprint coverage at different genomic regions
Download	• RPKM table	• Raw read count table
		• RPKM table
		• ORF annotation table
***Website compatibility***		
Applicability	• Desktop computers	• Desktop computers
		• Mobile devices

## DATABASE ADDITIONS AND ENHANCEMENTS

### Significant expansion of ribo-seq datasets

**Figure 1.**
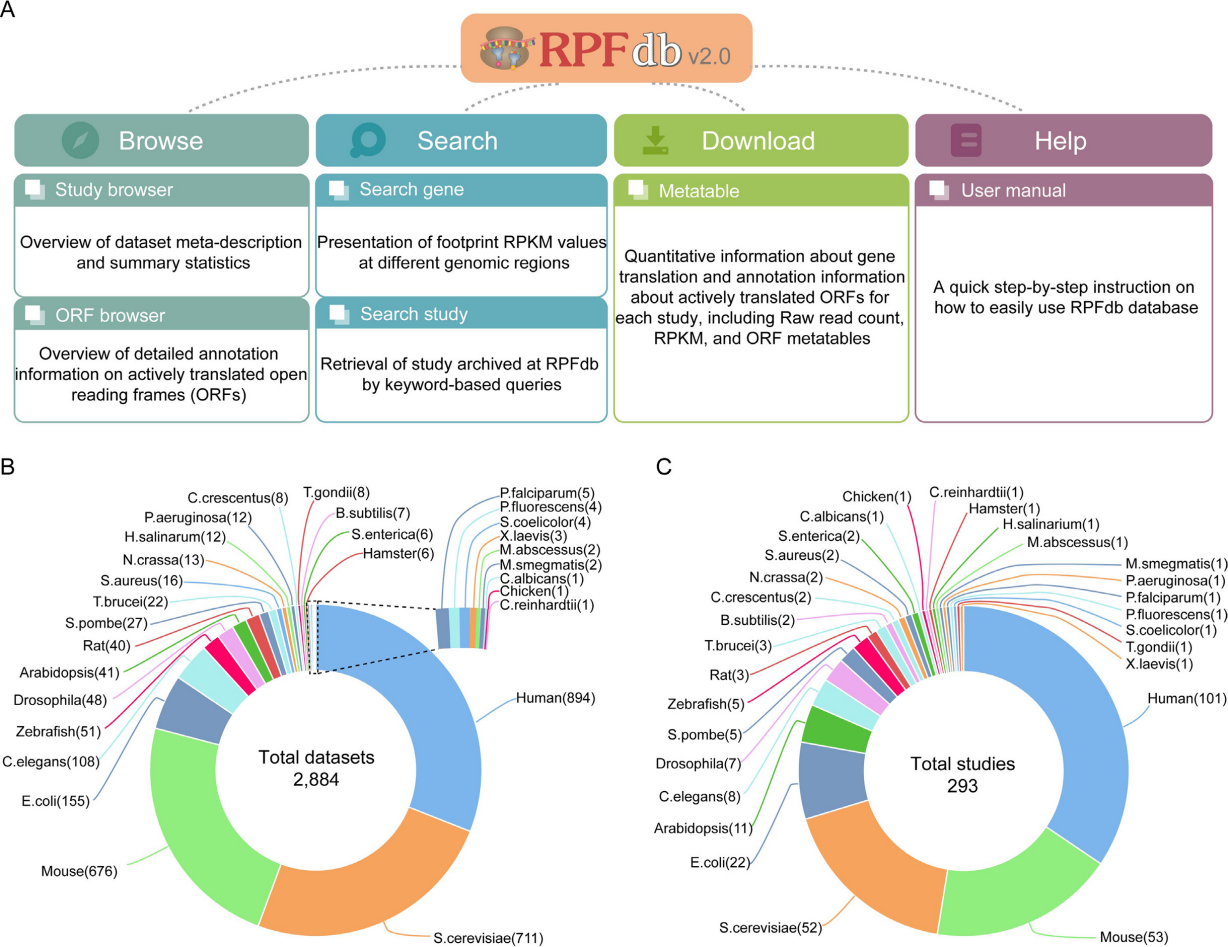
Overview of RPFdb v2.0. (**A**) Functional modules in RPFdb v2.0. (**B**) The number of ribo-seq datasets in species. (**C**) The number of studies in species.

To obtain a more comprehensive set of ribo-seq data, we have included more data sources. The SRA (13), the European Nucleotide Archive (ENA) (24) and DDBJ Sequence Read Archive (DRA) (25) were queried with keywords, including ‘ribosome profiling’, ‘ribo-seq’, ‘riboseq’, ‘ribosome protected fragments’, ‘ribosome footprints’, ‘ribosome footprinting’ and ‘RPF’. After manual screening, we retrieved additional 2107 ribo-seq datasets from 211 studies, bringing the current total number of available datasets to 2884 and the number of studies to 293 (Figure 1). Besides the eight species included in the previous version, 21 other species have been added, including *Bacillus subtilis, Chlamydomonas reinhardtii, Candida albicans*, Chinese hamster, *Caulobacter crescentus*, Chicken, *Halobacterium salinarum, Mycobacterium abscessus, Mycobacterium smegmatis, Neurospora crassa, Plasmodium falciparum, Pseudomonas aeruginosa, Pseudomonas fluorescens*, Rat, *Salmonella enterica, Schizosaccharomyces pombe, Streptomyces coelicolor, Staphylococcus aureus, Toxoplasma gondii, Trypanosoma brucei*, and *Xenopus laevis*. Notably among these species, human has shown the most dramatic increase not only in number of studies (from 27 to 101) but also in number of datasets (from 202 to 894) (Figure 1B and C). The other major species are mouse, *Saccharomyces cerevisiae* and *E. coli*. The rest of the species, particularly for the new included species, have only a limited number of datasets available. Nevertheless, the inclusion of these new species provides increased flexibility and enhanced options for a wide range of evolutionary and comparative studies.

### Pre-processing optimization of ribo-seq data

The complicated experimental procedures of ribo-seq may introduce some inherent noise in the output data, which requires careful filtering that will not only minimize errors introduced by contamination but also enhance data efficiency. In the initial version of RPFdb, we directly selected the first 26 nucleotides of each sequencing read for alignment. In this update, we made more considerations on data pre-processing. To avoid adapter interference with downstream analysis, the 3′ adapter sequences were manually extracted for each dataset either from the publications or from the corresponding MultiQC outputs (26). If present in the ends of sequencing reads, the linked adapter sequences were removed using Cutadapt (version 1.16) (27). To eliminate rRNA and tRNA contamination, rRNA and tRNA sequences were fetched for each species from ENSEMBL (28) and UCSC (29), and then removed after mapping by Bowtie2 (version 2.3.4.1) (30). Considering that high-quality footprints are expected to have a characteristic distribution of read-lengths reflecting the size of a translating ribosome on the RNA, footprints with non-typical size were further excluded, and only those with 25–34 nucleotides in length were kept after contaminant removal and alignment. It is indubitable that these improvements will achieve more precise quantification of gene translation levels and more accurate visualization of gene translation. In addition, a number of quality control assessment metrices are provided to all the datasets for each study, including detailed FastQC reports, plots of read size and reading frame distributions, as well as P-site offset and enrichment of RPF reads in different genomic regions. These features will give users a better idea about the datasets before utilizing the data.

### New contents presented by ribo-seq analysis

Recent advances in global translatome analyses have demonstrated that a much larger proportion of genomes have protein-coding potential than originally thought, revealing the existence of numerous alternative ORFs (altORFs) in addition to annotated protein coding sequences (31,32). These altORFs include upstream ORFs (uORFs) in the 5′ untranslated regions, downstream ORFs (dORFs) in the 3′ untranslated regions, and long non-coding derived ORFs (lncORFs). Some of them have been shown to actively undergo translation (15,18,33–35), which may play critical roles in fine-tuning translation program by serving as either translational repressors or dampeners (36,37). Hence, in this update we performed a systematic detection of actively translated regions by RibORF (34). To increase the power of footprint signal, we combined ribo-seq data with duplicate samples. To reduce the false positive rate in ORF finding, we only used those footprints with clear sub-codon phasing or triplet periodicity to predict actively translated ORFs. In total, we predicted canonical and non-canonical translated ORFs for 1405 aggregated ribo-seq datasets. More detailed information on each ORF entry is provided, such as genomic position, strand, annotated category and encoded amino acid length. The inclusion of these information in our database will facilitate systematic characterization, functional exploration and downstream analysis of ORFs.

### Enhanced user interface features

The addition of actively translated ORFs was required for the development of a new interface for browsing and searching them in our database. To this end, we developed a dual-interface webpage to achieve both browse and search functions, which was then integrated into the RPFdb. This new webpage allows users to easily browse ORF content and to quickly search for a specific entry. Users can specify species, study and experimental conditions of interest. In addition, the ORF browser webpage provides a built-in filter that can help users further narrow down the results. The returned results of ORFs are presented in tabular form, which are also available for download by clicking the download button on the page.

The significant changes in both new ribo-seq data and new contents have demanded an enhanced web-interface. To facilitate count-based analyses such as differential translation detection, the download webpage was modified to support retrieving matrix of raw read counts. To facilitate inspection of translation of non-coding genes, the gene search webpage was modified to provide RPKM values of non-coding genes. Moreover, to achieve a better compatibility view, all the pages were enhanced to support facile viewing of page contents on different terminals, particularly for mobile devices.

## CONCLUSIONS AND FUTURE PLAN

We presented RPFdb v2.0, an updated database for genome-wide information of translated mRNA generated from ribosome profiling, which is dedicated to providing more accurate, comprehensive and convenient way for translatome research. In this update, one of the most significant characteristics is that the scale of its collected ribo-seq data has been greatly expanded. Furthermore, a refined analysis pipeline has been applied for the pro-processing and alignment of ribo-seq data to make more precise quantification and visualization. In addition to providing quantitative information and intuitive visualization-oriented information about gene translation, RPFdb v2.0 now provides annotation information about actively translated ORFs. Moreover, more web features have been made available, including RPKM value of non-coding genes and matrix of raw read counts. The web interface has been also optimized for delivering an improved user experience.

RPFdb v2.0 will be periodically updated with the latest release of ribo-seq data. Also, we plan to include paired RNA-seq datasets with ribo-seq datasets in our database to facilitate downstream analysis such as translational efficiency analysis, considering that it is usually a common practice in ribosome profiling that the RPF and fragmented RNA would be profiled in parallel. Moreover, we will continue to expand the database contents based on newly developed computational frameworks and to fine-tune the database features and functionalities. In conclusion, with these additions and enhancements, RPFdb will continue serving as a valuable resource and make important contributions to the gene regulation community.

## DATA AVAILABILITY

RPFdb is publicly available at http://www.rpfdb.org or http://sysbio.sysu.edu.cn/rpfdb.
